# NAPAbench 2: A network synthesis algorithm for generating realistic protein-protein interaction (PPI) network families

**DOI:** 10.1371/journal.pone.0227598

**Published:** 2020-01-27

**Authors:** Hyun-Myung Woo, Hyundoo Jeong, Byung-Jun Yoon

**Affiliations:** 1 Department of Electrical and Computer Engineering, Texas A&M University, College Station, Texas, United States of America; 2 Department of Mechatronics Engineering, Incheon National University, Incheon, Republic of Korea; 3 TEES-AgriLife Center for Bioinformatics and Genomic Systems Engineering, Texas A&M University, College Station, TX, United States of America; 4 Computational Science Initiative, Brookhaven National Laboratory, Upton, NY, United States of America; University of Michigan, UNITED STATES

## Abstract

Comparative network analysis provides effective computational means for gaining novel insights into the structural and functional compositions of biological networks. In recent years, various methods have been developed for biological network alignment, whose main goal is to identify important similarities and critical differences between networks in terms of their topology and composition. A major impediment to advancing network alignment techniques has been the lack of gold-standard benchmarks that can be used for accurate and comprehensive performance assessment of such algorithms. The original NAPAbench (network alignment performance assessment benchmark) was developed to address this problem, and it has been widely utilized by many researchers for the development, evaluation, and comparison of novel network alignment techniques. In this work, we introduce NAPAbench 2—a major update of the original NAPAbench that was introduced in 2012. NAPAbench 2 includes a completely redesigned network synthesis algorithm that can generate protein-protein interaction (PPI) network families whose characteristics closely match those of the latest real PPI networks. Furthermore, the network synthesis algorithm comes with an intuitive GUI that allows users to easily generate PPI network families with an arbitrary number of networks of any size, according to a flexible user-defined phylogeny. In addition, NAPAbench 2 provides updated benchmark datasets—created using the redesigned network synthesis algorithm—which can be used for comprehensive performance assessment of network alignment algorithms and their scalability.

## Introduction

Comparative network analysis through local or global network alignment provides effective computational means to identify orthologous proteins and conserved functional modules (*e.g*., molecular complexes or pathways) across biological networks of different species. It also enables transferring prior knowledge of a well-studied species to a less-studied species, potentially leading to significant savings in terms of experimental cost and time [1]. However, one of the major barriers slowing down further advances in comparative network analysis research has been the lack of a gold standard benchmark that allows a fair and comprehensive performance assessment of comparative network analysis algorithms. To overcome this barrier, NAPAbench (Network Alignment Performance Assessment benchmark)—probably the first comprehensive synthetic benchmark for network alignment—was released in 2012 [2]. The original NAPAbench is comprised of three suites of benchmarks, for testing pairwise, 5-way, and 8-way alignment, respectively. Each suite consists of three different datasets generated by different network synthesis models (*i.e*., DMC, DMR, and CG), where each dataset contains ten network families generated independently by a given synthesis model. Since the original release, NAPAbench has been widely used for evaluating the performance of various network alignment algorithms [3–14].

However, the key parameters of the network synthesis models that were used in the original NAPAbench were trained based on the PPI networks in Isobase [15], which was released in 2010. Due to the advances in high-throughput profiling and text mining techniques, the quality and coverage of the latest PPI networks have been dramatically improved during the past decade. As a result, the latest real PPI networks contain many new proteins and a significantly larger number of interactions and they tend to be much denser compared to the networks in the original NAPAbench. In order to keep pace with the recent developments, we introduce NAPAbench 2 in this paper. NAPAbench 2 consists of benchmarks that consist of families of networks generated by new and/or updated network synthesis models, whose characteristics closely resemble those of the latest real PPI networks. The new release of NAPAbench is also accompanied by a network synthesis tool with an intuitive and user-friendly interface, which allows users to easily create additional benchmarks that consist of network families with an arbitrary number of networks of any size, according to a user-specified phylogeny.

## Materials and methods

### Dataset and preprocessing

In order to learn parameters of the network synthesis models in NAPAbench 2, we analyzed characteristics of the latest real PPI networks in terms of topological structure and biological correspondence between proteins in different PPI networks. We used STRING database (v10.0) [16] to analyze the key properties of the real PPI networks as it provides comprehensive coverage and rich source of proteins by integrating a number of public PPI network databases: BIND [17], DIP [18], GRID [19], HPRD [20], IntAct [21], MINT [22], and PID [23]. Among various eukaryotes, we selected five species as our references: human (*H. sapiens*), yeast (*S. cerevisiae*), fly (*D. melanogaster*), mouse (*M. musculus*), and worm (*C. elegans*).

First, to study the topological structure of the PPI networks, we only employed direct protein interactions (i.e., protein binding) and retained reliable protein interactions that have been experimentally validated with a confidence score greater than 400 (i.e., the medium level of confidence recommended by STRING). Since the aforementioned filtering steps made the networks fragmented, we extracted the largest connected subnetwork from each of the networks and utilized them as our reference networks. Table 1 shows differences in the number of edges and proteins between the reference PPI networks from STRING and Isobase.

**Table 1:** The number of edges and proteins in real PPI networks from Isobase and STRING.

	Isobase		STRING	
Species	# of Edges	# of Proteins	# of Edges	# of Proteins
*H. Sapiens*	34,250	8,580	95,095	11,852
*S. Cerevisiae*	27,981	4,899	88,312	5,724
*D. Melanogaster*	19,579	6,572	64,929	6,652
*C. Elegans*	4,211	2,511	60,234	6,590
*M. Musculus*	23	16	112,321	10,125

**Fig 1:**
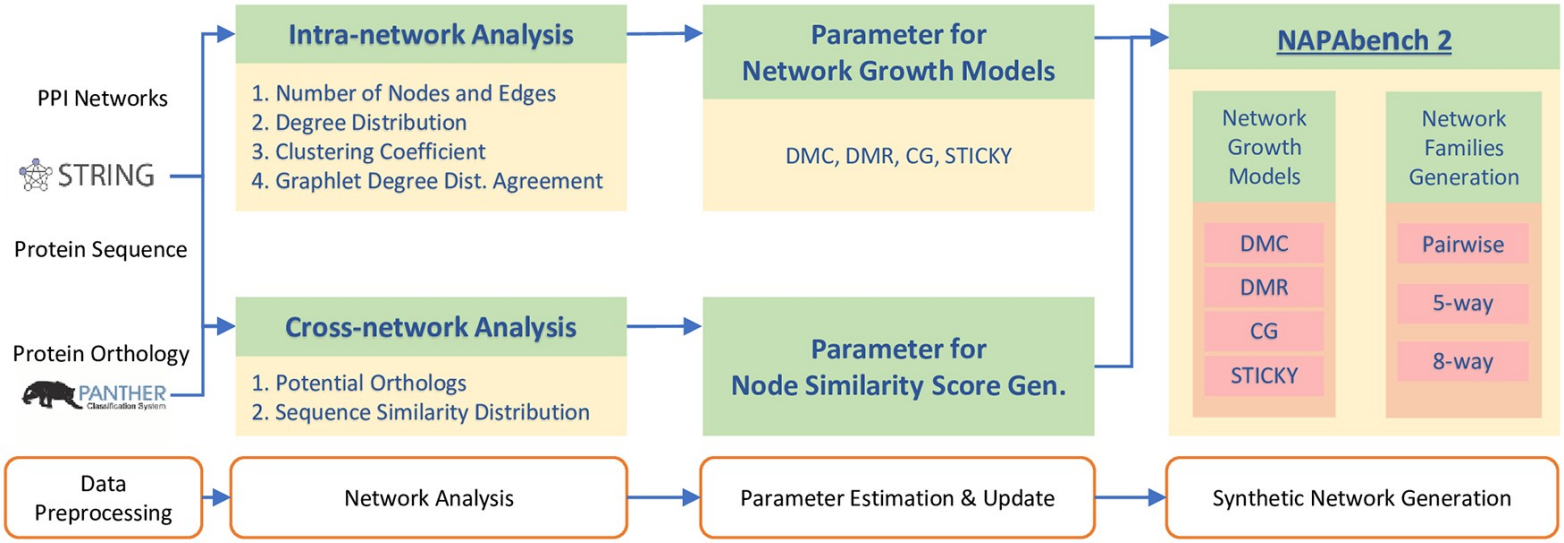
NAPAbench 2 analysis and network family generation procedure.

Then, we observed the distribution of protein sequence similarity scores of each reference network pair to analyze the biological correspondence between proteins in the different PPI networks. To do this, we downloaded protein sequences (i.e., amino acid sequences) of the five species from the STRING database and computed amino acid sequence similarity score using BLASTp [24] between nodes that belong to different networks. For a given node pair, if it has multiple BLAST bit scores, we took the highest bit score as a representative similarity score. Additionally, we excluded BLAST bit scores whose e-value is greater than 0.01. PANTHER orthology annotation [25] was used to determine the protein orthology between proteins in different species. Note that NAPAbench 1 utilized KEGG orthology (KO) group annotations [26, 27] as a reference. Both databases have been manually curated by experts and widely utilized in diverse protein studies [28]. An overall procedure is shown in Fig 1.

### Statistical feature analysis of real PPI networks

To synthesize realistic benchmark network families, it is necessary to select features capturing key characteristics of the PPI networks. For this purpose, we categorized the features from two different perspectives: i) intra-network features catching the topological structures of the PPI networks and ii) cross-network features detecting the biological relevance of proteins in different PPI networks. As intra-network feature analysis, we utilized graphlet degree distribution agreement (GDDA) [29] in addition to the degree distribution and clustering coefficient that were utilized in the original NAPAbench. For the cross-network feature analysis, we compared distributions of the BLAST bit scores for orthologous/non-orthologous protein pairs in different networks. To accomplish this, we employed PANTHER protein orthology annotation as it has been manually well-curated by a group of experts since it was released in 2010 [25].

#### Intra-network feature analysis

**Fig 2:**
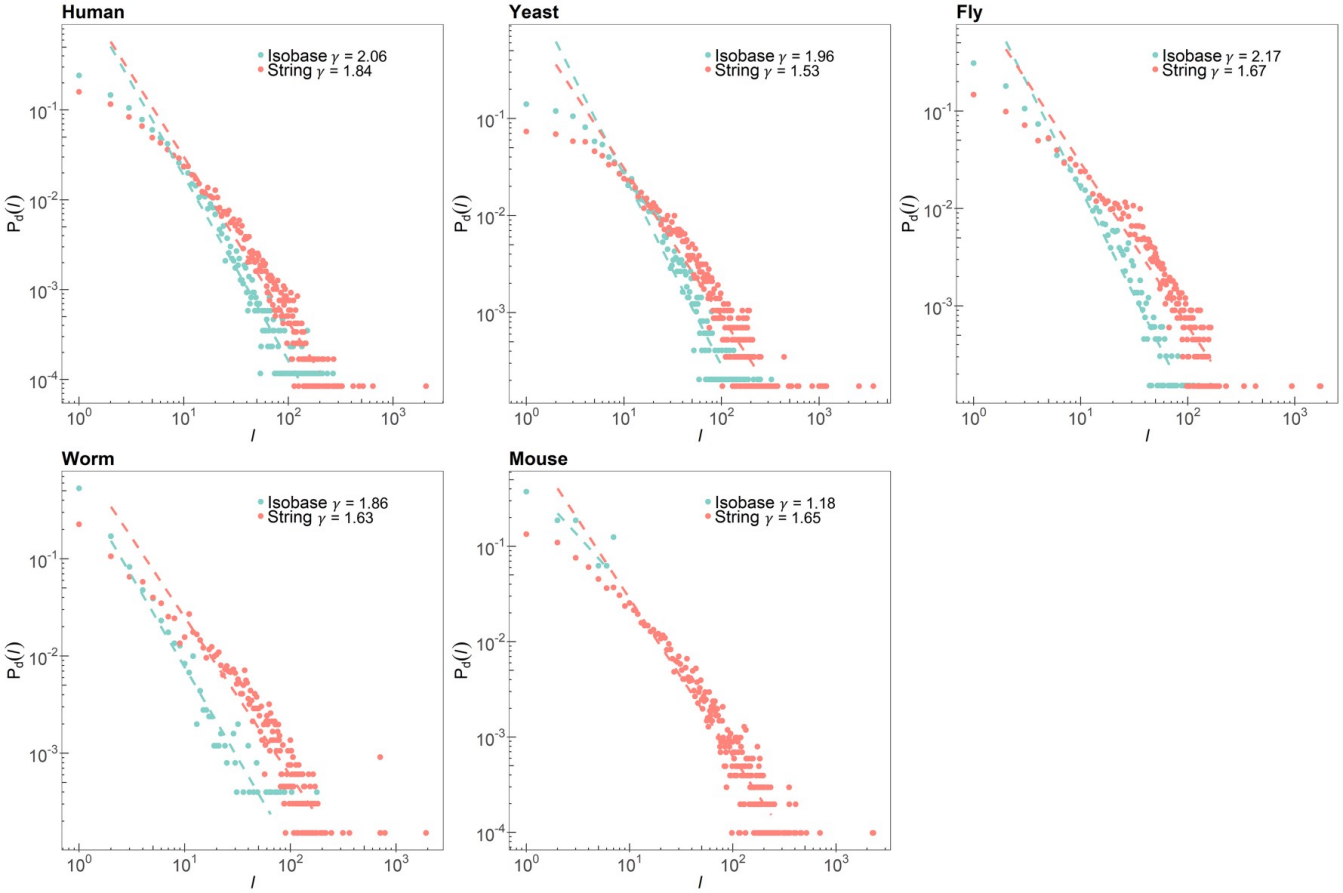
Node degree distribution of the five species in STRING and Isobase.

As a feature capturing global topological structures, we first investigated individual degree distribution of each PPI network in STRING and Isobase. For a given node, the node degree is defined as the number of edges (i.e., interactions) connected to the node. We assumed that a PPI network can be modeled as a scale-free network following the power-law degree distribution [30]. In other words, for a given node, the probability that the node has a degree *k* is given by *P*_*d*_(*k*) ∼ *k*^−*γ*^, where *γ* is a degree exponent [2]. Note that the degree exponent tends to be smaller as the network has more number of nodes with higher node degree. We hypothesized that the PPI networks in STRING have more proteins with higher node degrees compared to those of Isobase as novel proteins and their interactions have been identified and archived in the public databases over the past decade in accordance with the rapid advances of high-throughput profiling techniques. Fig 2 shows the degree distributions and corresponding estimated degree exponents for the five species in STRING and Isobase. The degree exponents were estimated through the linear regression function polyfit in MATLAB. The degree exponents for Isobase ranged from 1.86 to 2.17 and ranged from 1.53 to 1.84 for STRING, respectively. As we expected, the PPI networks in STRING had more proteins with higher node degrees, which resulted in smaller degree exponents. In fact, hub nodes, nodes with higher node degrees, play crucial roles in a scale-free network as they not only provide the shortest paths to distant nodes within subnetworks, but also characterize the topological features of the network. Based on the analysis of the degree distributions, we found out that a degree exponent is a discriminate feature recapitulating global topological structures. Note that we excluded a degree exponent estimated from a mouse PPI network from our analysis since the size of the network (i.e., the number of nodes) was too small to be compared with those of other PPI networks.

**Fig 3:**
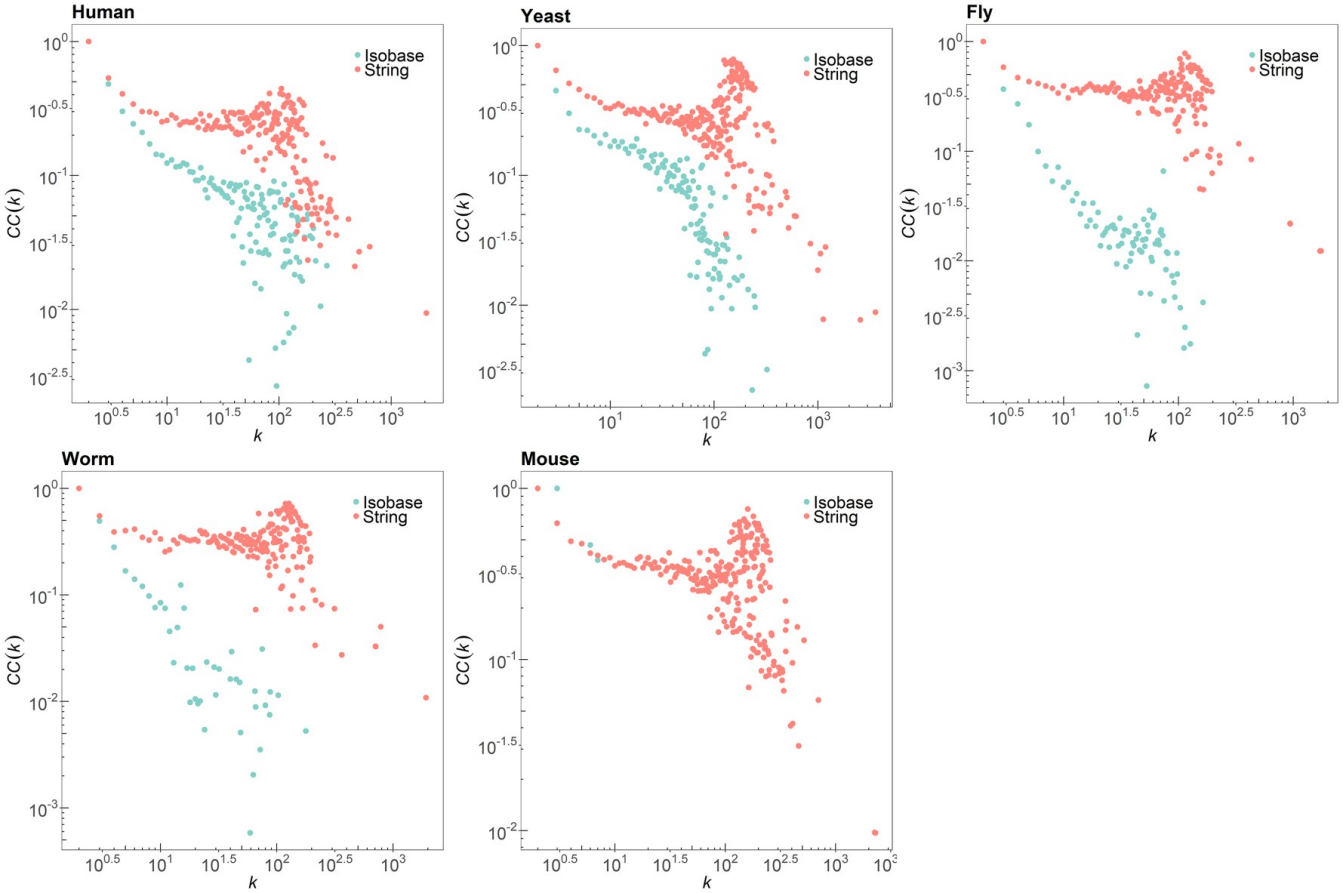
Clustering coefficient distribution of the five species in STRING and Isobase.

Next, we observed individual distribution of local clustering coefficients of each PPI network as a feature capturing local structures of the networks. Given a node *v* with a degree *k*, the clustering coefficient is defined as $CC\left(k\right)=\frac{2e}{k(k-1)}$, where *e* is the number of connections among the neighbors of *v*. The clustering coefficient of a node indicates how close the given node and its neighborhood are for forming a complete graph, a clique. In fact, proteins in a functional subnetwork of a PPI network tend to be densely connected to each other while sparsely connected to nodes outside the subnetwork [31]. Therefore, if a PPI network contains a large number of proteins with high clustering coefficients, the network is more likely to have an increased number of functional subnetworks. Fig 3 shows the comparison results between the clustering coefficient distributions of the PPI networks in STRING and Isobase. It shows that the PPI networks in STRING have more nodes with high clustering coefficients than those of Isobase, meaning that the latest PPI networks from STRING could have more functional subnetworks than the PPI networks from Isobase. These results clearly support the necessity of new benchmark datasets reflecting the local topological features of the latest real PPI networks.

In addition to the features aforementioned, we considered a graphlet degree distribution as a new feature to capture the detailed local interaction patterns as well as the statistical global PPI network structure [29]. The graplets are 29 small connected induced subgraphs consisting of 2 to 5 nodes, respectively, and there are 73 automorphism orbits within the graphlets according to topological relevance. For a given PPI network G, we can generate the set of 73 graphlet degree distributions {$D_{G}^{j}(k)$}, where the distribution $D_{G}^{j}(k)$ for *j*-th orbit is defined as the number of nodes touching *k* *j*-th orbits. Note that $D_{G}^{0}(k)$ is a degree distribution. As graphlet degree distributions rigorously detect not only local interaction patterns around focal nodes but also global structure of the PPI network, a number of PPI network alignment algorithms have been proposed based on the graphlet degree distributions [8, 32–34]. Note that we utilized a graphlet degree distribution agreement (GDDA) score between two networks as the new feature indicating degree of similarity of local and global topological interaction patterns to learn the parameters of each network growth model.

#### Cross-network feature analysis

As a cross-network feature, we analyzed the orthology relationship between proteins in different PPI networks thereby learning core parameters of NAPAbench 2 to synthesize realistic network families through the network growth models. To do this, we followed the similar procedure presented in the original NAPAbench. Hence, we here briefly introduce the overall procedure for the cross-network feature analysis.

**Fig 4:**
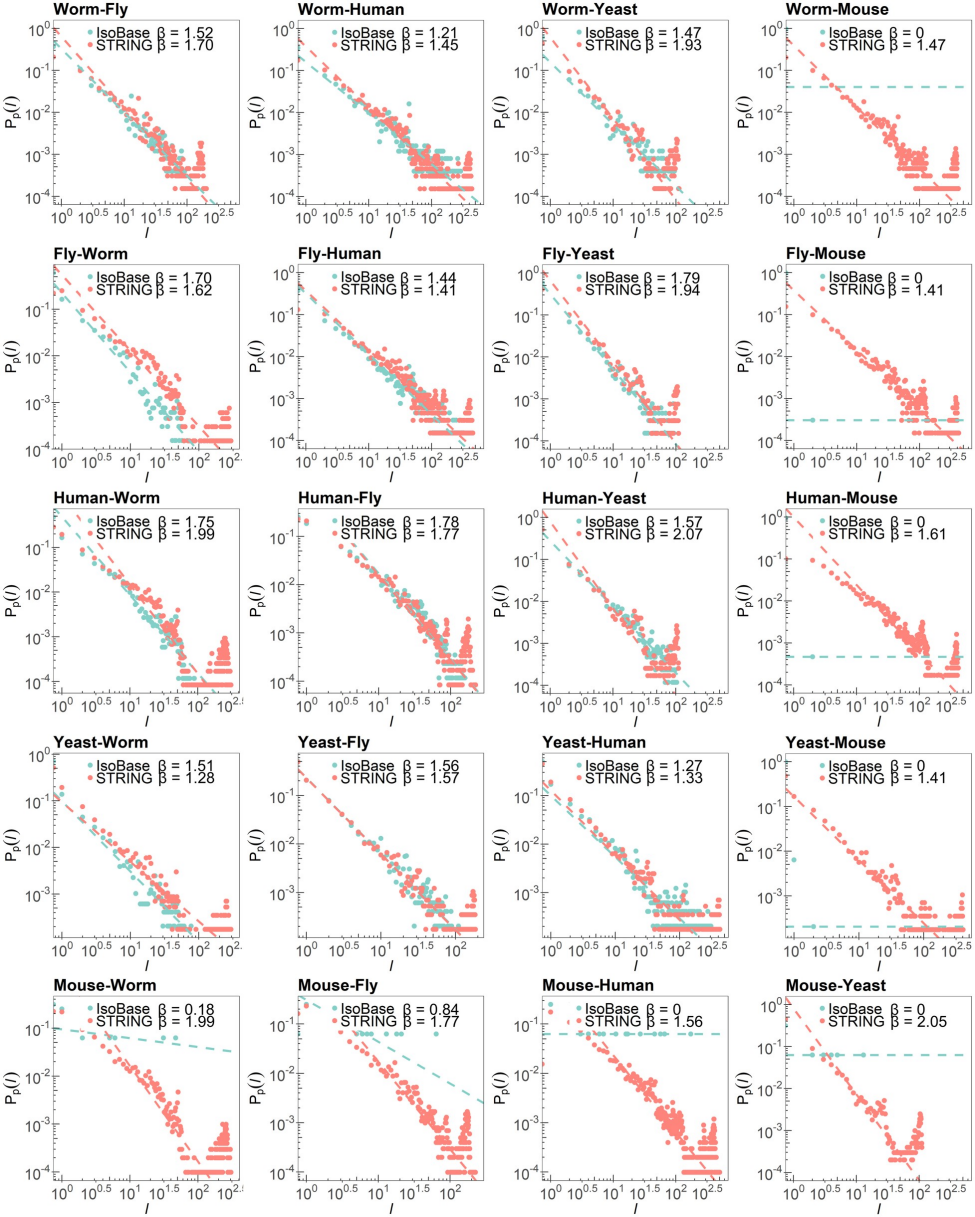
Distributions of the number of the potential orthologous nodes between different PPI network pairs. Note that the mouse PPI network in Isobase results in significant errors because it contains a large number of singleton nodes.

Suppose that we have two PPI networks $G_{1}=(V,E)$ and $G_{2}=(U,D)$, where V and U represent sets of nodes (i.e., proteins) and E and D indicate sets of edges (i.e., protein interactions) in each network. We analyzed the number of potential orthologous proteins and their similarity scores (i.e., BLAST bit scores) across different networks. That is, given a node v∈V, we estimate the number of proteins u∈U that are potentially orthologous to the protein *v*. We assumed that a protein pair from different PPI networks is highly likely to be orthologous if they have a high sequence similarity score. In other words, given a node v∈V, we estimate potential orthologous proteins as follow:

$$\begin{array}{c} N(v)=|\{u|u\in U,s(v,u)>T_{s}\}|, \end{array}$$ (1)

where we set the threshold *T*_*s*_ as 45. We defined *P*_*p*_(*l*) as a probability density function that the protein node *u* in $G_{1}$ has *l* potential orthologous nodes in the network $G_{2}$. Similar to the analysis of the degree distribution in the previous section, we assumed that the distribution *P*_*p*_(*l*) can be modeled by a power-law distribution *P*_*p*_(*l*) ∼ *l*^−*β*^, and used the polyfit function in MATLAB to estimate the exponent *β*. Fig 4 shows the comparison results between the estimates of the *β* of PPI networks in STRING and Isobase. Based on the linear regression results, we observed that STRING had the exponent *β* ranged from 1.28 to 2.07, and the exponent ranged from 1.27 to 1.79 for Isobase. Interestingly, the PPI networks in STRING had lager *β* compared to those of PPI networks in Isobase, meaning there were more node pairs with low similarity scores but fewer node pairs with high similarity scores. However, we observed a peak at the high sequence similarity score region (greater than 10^2^) in the potential orthologous node distribution of PPI network pairs in STRING as shown in Fig 4. These results show that although the regression analysis may not clearly capture the peak in the high sequence similarity regions, there is higher chance to have a larger number of high sequence similarity protein pairs in PPI networks obtained from STRING.

**Fig 5:**
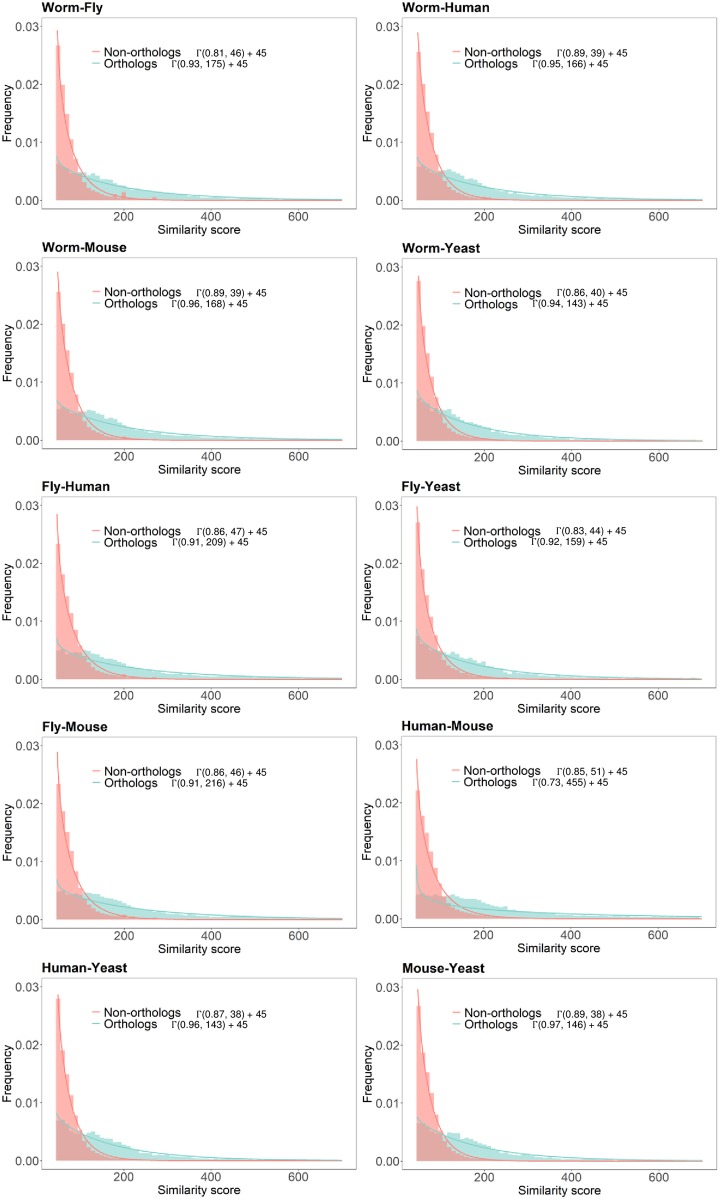
Estimation of the shape and scaling parameters of the sequence similarity score distributions for different PPI network pairs in STRING.

**Fig 6:**
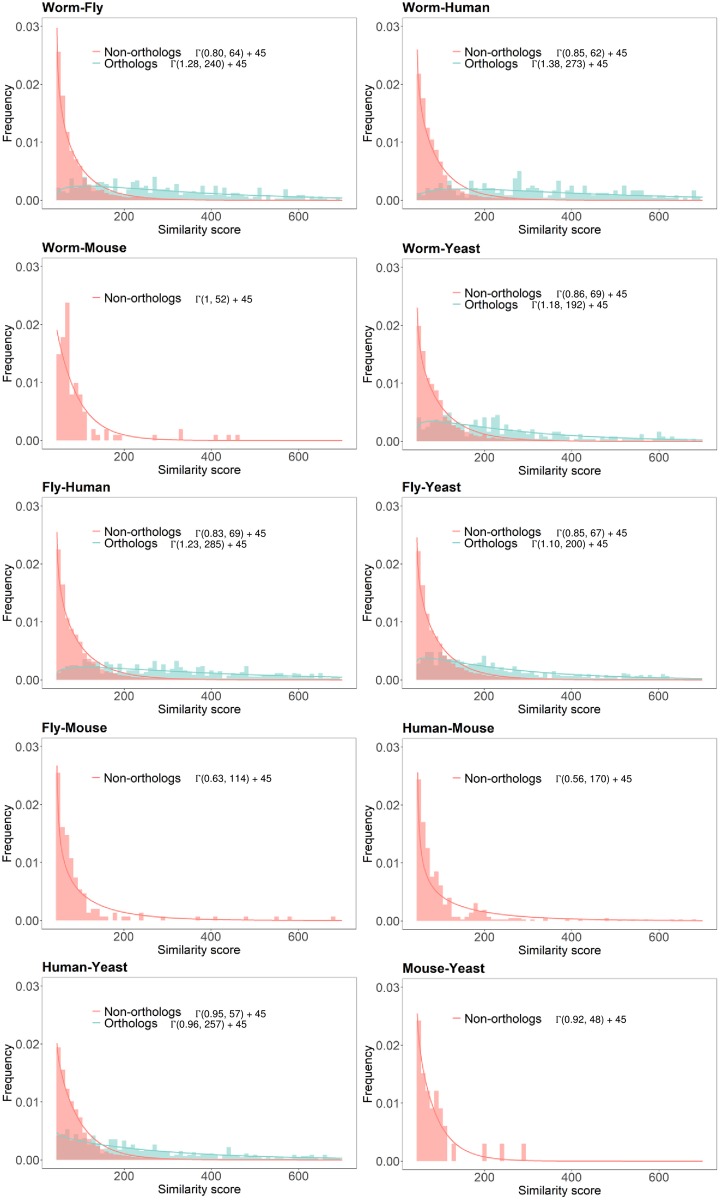
Estimation of the shape and scaling parameters of the sequence similarity score distributions for different PPI network pairs in Isobase.

Next, for each real PPI network pair, we estimated the distribution of the BLAST bit scores for orthologous protein pairs as well as that for the non-orthologous pairs. As previously noted in [2], the distribution can be effectively modeled as a Gamma distribution *X* ∼ Γ(*κ*, *θ*), where *κ* is a shape parameter and *θ* is a scaling parameter. We estimated the shape and scaling parameters through the curve fitting function fitdist in MATLAB. Figs 5 and 6 show the analysis results of PPI networks in STRING and Isobase, respectively. For STRING, the estimated scaling and shape parameters of orthologous protein pairs ranged from 0.91 to 0.97 and from 143 to 216, respectively. These parameters significantly differ from those of network pairs in Isobase, where the scaling and shape parameters ranged from 0.96 to 1.38 and from 192 to 284, respectively. Note that we excluded the parameters estimated between human and mouse in STRING from our analysis as they were outliers. In addition, we removed the parameters estimated between worm and mouse; fly and mouse; human and mouse; and mouse and yeast PPI networks in Isobase from our analysis since homologous protein pairs did not exist. For non-orthologous protein pairs, the scaling and shape parameters of PPI networks in STRING ranged from 0.81 to 0.89 and 38 to 47, respectively, and they ranged from 0.56 to 1 and 48 to 170 in Isobase, respectively. The clear differences also support that the synthesized networks in the original NAPAbench have been outdated and it clearly motivates the necessity of updating the network synthesis models and the benchmark datasets.

## Overview of network synthesis models

NAPAbench 2 provides rich benchmark datasets based on four different network growth models: DMC (duplication-mutation-complementation) [35], DMR (duplication with random mutation) [36], CG (crystal growth) [37], and STICKY [38] models. The first three network models were introduced in the original NAPAbench [2] and we newly included the STICKY model in this release since the STICKY model accurately captures critical features of the real PPI networks such as the graphlet degree distribution [39, 40]. In the STICKY model, for the given number of nodes, interaction patterns between proteins are formed in a way that two proteins with a higher stickiness index can have a higher chance to interact with each other. Note that we extended the STICKY model in order to accommodate it into the NAPAbench 2 framework performing a bifurcation process according to a given phylogenetic tree to synthesize a set of biologically related network families. Different from the original STICKY model, where it assigns stickiness index to all protein nodes and produces all interactions simultaneously, the extended STICKY model is capable of gradually forming interaction patterns in accordance with the increase in its size (i.e., the number of nodes). Since the first three networks growth models (i.e., DMC, DMR, and CG models) were described in the original NAPAbench paper [2], we omit the detailed steps of the models regarding extending its size and forming interactions and we describe the procedure of the STICKY model and its extended version that has been adopted in the NAPAbench 2.

### STICKY model

Let G=(V,E) be the graph representing a given PPI network, where V is a set of nodes (proteins) $V=\{v_{1},v_{2},...,v_{n}\}$ and edges $E=\{e_{i,j}\}$ indicating interactions between a protein *v*_*i*_ and *v*_*j*_. STICKY model generates a synthetic network based on the following procedures:

STEP 1 Given *N* nodes in the network, it assigns an initial node degree *d*_*i*_ to each node *v*_*i*_.
STEP 2 For each node *v*_*i*_, it assigns a stickiness index based on the following equation: $\theta_{i}=d_{i}/\sqrt{\sum_{j=1}^{N}d_{j}}$.
STEP 3 For every node pair, it inserts edge between *v*_*i*_ and *v*_*j*_ if *x* ≤ *θ*_*i*_ ⋅ *θ*_*j*_, where *x* is a sample drawn from a uniform distribution *U*(0, 1).

### Extended STICKY model

As we described earlier, NAPAbench performs a bifurcation process along a given phylogenetic tree in order to synthesize a set of realistic PPI network families that are biologically related to each other. More specifically, as the bifurcation process proceeds along the phylogenetic tree, the network model constructs subsequent networks by extending PPI networks from the ancestor PPI network on the tree. To adapt the static STICKY model in the NAPAbench framework, we extended the STICKY model in a way that it can iteratively add a new node to itself until the number of nodes in the network reaches the predefined size as follows:

STEP 1 Given a PPI network with *N* nodes, we introduce a new node *v*_*N*+1_ and assign a node degree *d*_*N*+1_ drawn from a power-low distribution *p*(*d*) = *cd*^−*γ*^. We set the degree exponent *γ* to 1.6 based on our analysis.
STEP 2 For each node *v*_*i*_, *i* = 1, 2, …, *N* + 1, we update the corresponding sticky index *d*_*i*_ as follow: $\theta_{i}=d_{i}/\sqrt{\sum_{j=1}^{N+1}d_{j}}$.
STEP 3 For each node pair (*v*_*N*+1_, *v*_*j*_), where *j* = 1, 2, …, *N*, we insert edge between *v*_*N*+1_ and a neighbor node *v*_*j*_ if *x*_*j*_ ≤ *θ*_*N*+1_ ⋅ *θ*_*j*_, where *x*_*j*_ is a sample drawn from a uniform distribution *U*(0, 1).
STEP 4 The extended STICKY model randomly removes an edge between *v*_*N*+1_ and a neighbor node *v* ∈ *Neighborhood*(*v*_*N*+1_) according to a user-defined probability *f*_*del*_.
STEP 5 We randomly select a node among the neighborhood *v* ∈ *Neighborhood*(*v*_*N*+1_) based on a distribution of STICKY indices and assign the biological function of the selected adjacent node to the node *v*_*N*+1_.
STEP 6 *N* is increased by 1, *N* ⇐ *N* + 1, and we repeat STEP 1 through 6 until the network reaches the target size (i.e., the number of proteins in the network).

### Parameter estimation of NAPAbench 2

In NAPAbench 2 framework, there are two types of parameters: intra-network parameters and cross-network parameters. The intra-network parameters are dependent on the network growth models. In other words, each network growth model in the NAPAbench 2 has its own parameters affecting a topological structure of the synthetic networks. The cross-network parameters are independent of the network growth models and they determine biological correspondence of node pairs in different synthesized networks.

For the intra-network parameters, we optimized the parameters of each network growth model through grid search so that they can generate realistic PPI network families whose features closely resemble those of the real networks observed from the intra-network feature analysis of the PPI networks in STRING. First, for each network model, we divided range of the parameters ranging [*p*_*min*_, *p*_*max*_] by the equal width 0.05. Next, we generated synthetic networks based on each parameter combination and selected the best parameter combinations, where it results in the best fitting to our analysis of the PPI networks in STRING in terms of degree distribution, clustering coefficient distribution, the number of nodes and edges, and GDDA score. Through the grid search, we optimized the parameters as follows: *q*_*con*_ = 0.5 and *q*_*mod*_ = 0.4 for DMC, *q*_*new*_ = 0.85 and *q*_*del*_ = 0.4 for DMR, and *s*_*del*_ = 0.55 and *s*_*f*_ = 50 for STICKY model. Note that we did not adjust the parameter *δ* = 4 for the CG network growth model due to the fact that the model was sensitive to changes in the parameter, generating unrealistic results with other values.

We tuned the cross-network parameters according to the results of cross-network feature analysis. With regard to parameters of node similarity score generation for the orthologous protein pairs, we took the average of the scaling and shape parameters, yielding *k*_*o*_ = 0.94 and *θ*_*o*_ = 169.49, and for the non-orthologous proteins pairs, we used *k*_*n*_ = 0.86 and *θ*_*n*_ = 42.00. A probability of not assigning the null function to a given node $P_{f_{O}}$ was set to 0.9 and a random scaling factor of similarity score λ_*max*_ was set to 0.1, respectively.

### Construction of benchmark datasets through updated model parameters

In the NAPAbench 2, we adopted the same procedure utilized in the original NAPAbench to generate synthetic benchmark datasets. Hence, we briefly introduce the overview of the synthetic network generation process described in [2]. Suppose that we generate a family of *N* synthetic PPI networks $G=\left\{G_{1},G_{2},\ldots ,G_{N}\right\}$. Each network $G_{k}=(V_{k},E_{k},F_{k})$ consists of a set $V_{k}=\left\{v_{k,1},v_{k,2},\ldots ,v_{k,N_{k}}\right\}$ of *N*_*k*_ nodes; a set $E_{k}=\left\{e_{k,ij}\right\}$ of *M*_*k*_ edges, where *e*_*k*,*ij*_ denotes the edge between nodes *v*_*k*,*i*_ and *v*_*k*,*j*_; and a set $F_{k}=\left\{f_{k,1},f_{k,2},\ldots ,f_{k,N_{k}}\right\}$ which maps each node *v*_*k*,*i*_ to a functional group *f*_*k*,*i*_ in FO={F0,F1,F2,…}, a set of all functional orthology (FO) annotations.

In order to synthesize a set of PPI networks biologically related to each other, we utilized a bifurcation process over a phylogenetic tree T, where it has a single root node and each parent node has exactly two child nodes. That is, starting from the root node corresponding to the ancestor network $G_{S}$, we repeated a bifurcation process until the phylogenetic tree is developed to have *N* leaf nodes corresponding to *N* synthetic networks. In each bifurcation process, since each internal node in the phylogenetic tree T has exactly two child nodes, we first duplicate the parent network and inherent the functional annotations. Then, each duplicated network corresponding to the child node starts an independent network extension process based on the predefined network growth model by a user. When developing the networks, we assigned the node similarity scores based on the Gamma distribution with the parameters learned from our analysis. We repeated the aforementioned process until it generates *N* synthetic networks. Note that we obtained the root network $G_{S}$ by developing a small seed network $G_{seed}$, and we utilized the different seed networks according to the network growth models. To generate the root network $G_{S}$ for each network growth model, we utilized the same seed networks adopted in the original NAPAbench and we used the seed network for the CG model as the seed network for the STICKY model. Note that network growth models can generate a singleton node, where it is completely isolated from others because the edge perturbation steps in each network growth model can remove all edges connecting to the newly added node. If it generates a singleton node, we discard the singleton node and repeat the node generation procedure until a non-singleton node is generated.

### Graphical user interface of NAPAbench 2

**Fig 7:**
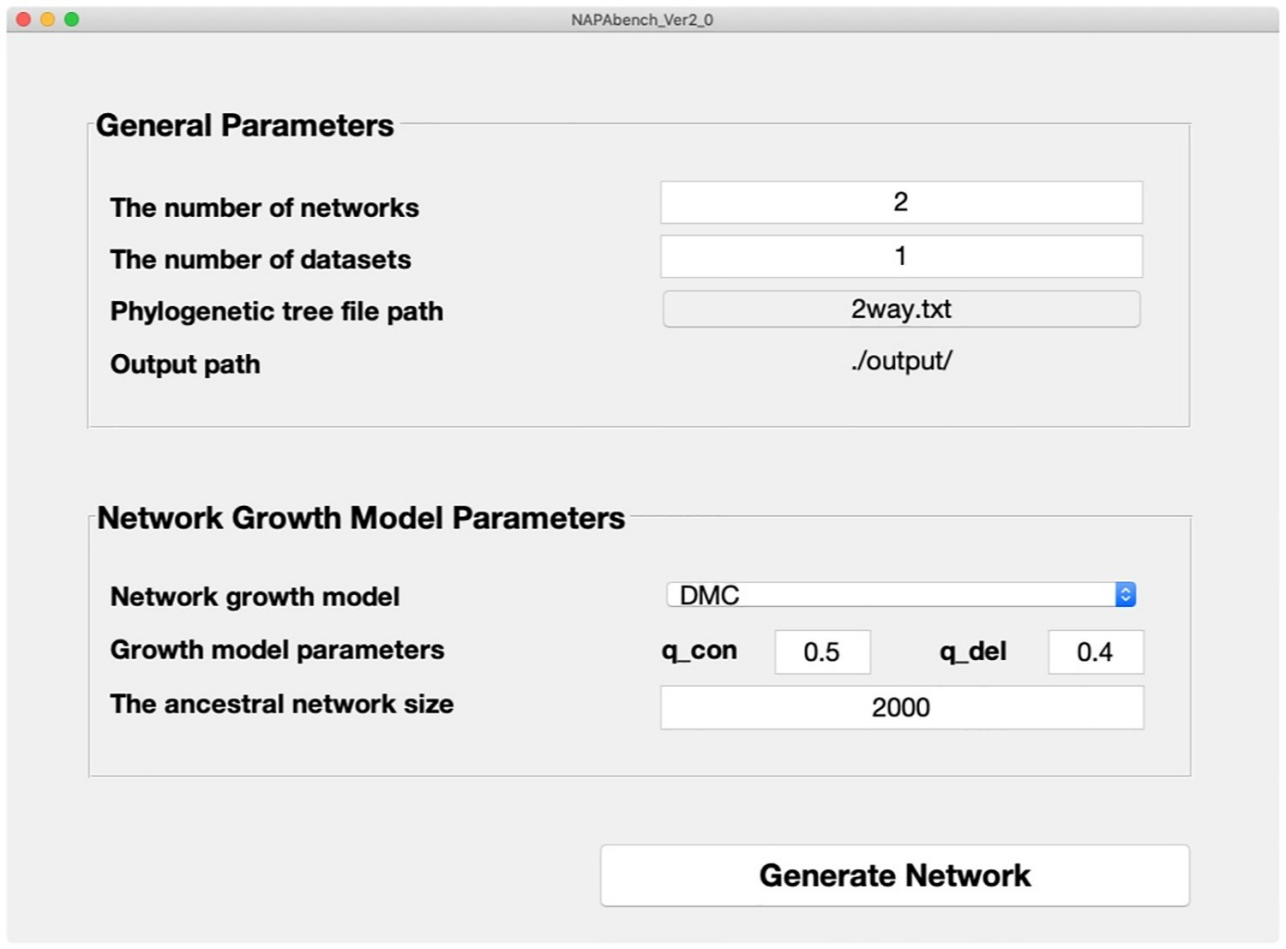
Graphical user interface (GUI) of NAPAbench 2 network synthesis tool.

The original NAPAbench was released as a command line-based standalone toolkit which makes it difficult for users to generate new benchmark datasets according to their preference. To enhance usability, we implemented the graphical user interface (GUI) for NAPAbench 2 as shown in Fig 7. We believe that the GUI implementation clearly lowers the hurdle for users to generate new benchmark datasets based upon their own needs. Additionally, we also provide the default parameter settings for the pairwise, 5-way, and 8-way network families along with phylogenetic tree files that were utilized to construct the standard benchmark datasets of NAPAbench 2. The supplementary material provides the detailed guidelines.

## Results

Based on the extensive intra-network and cross-network analysis, we updated the key model parameters for each network growth model in NAPAbench 2. In the following subsection, we compared topological structures of the synthetic networks against that of real PPI networks in order to verify the topological similarity between synthetic and the real PPI networks.

### Comparison of synthetic networks to real PPI networks in STRING

We carried out experiments to compare the statistical differences across the networks generated by the network growth models in NAPAbench 2, the networks generated by the models in NAPAbench 1, and the real PPI networks in STRING. For this purpose, we first generated 100 synthetic networks, each of which containing 5, 500 nodes, for each network growth model in NAPAbench 1 and 2. Then we counted the total number of edges and computed the average node degree for the 100 realizations of synthetic networks generated by each network model. As shown in Table 2, the average node degrees of human, yeast, and fly PPI networks were 8.02, 15.42, and 9.76, respectively, which were significantly higher than those of the 100 network realizations generated by models in NAPAbench 1. On the other hand, DMC, DMR, and STICKY models in NAPAbench 2 generated PPI networks whose average node degrees were 9.83, 12.12, and 8.40, respectively. This comparison clearly shows that the network growth models in NAPAbench 2 are able to synthesize network families whose average edge densities are noticeably closer to those of the latest PPI networks. Note that because the parameter of the CG model in NAPAbench 2 has not been updated, the average node degree of 100 realizations generated by the CG model remained the same as that of the CG model in NAPAbench 1.

**Table 2:** Comparison of statistics of synthetic networks with real PPI networks.

	STRING			NAPAbench 2				NAPAbench 1		
	Human	Yeast	Fly	DMC	DMR	CG	STICKY	DMC	DMR	CG
# of nodes	11,852	5,724	6,652	5,500						
# of edges	95,095	88,312	64,929	54,052	66,650	21,986	46,214	11,241	11,156	21,985
Edges/Node	8.02	15.43	9.76	9.83	12.12	4	8.40	2.04	2.03	4

**Fig 8:**
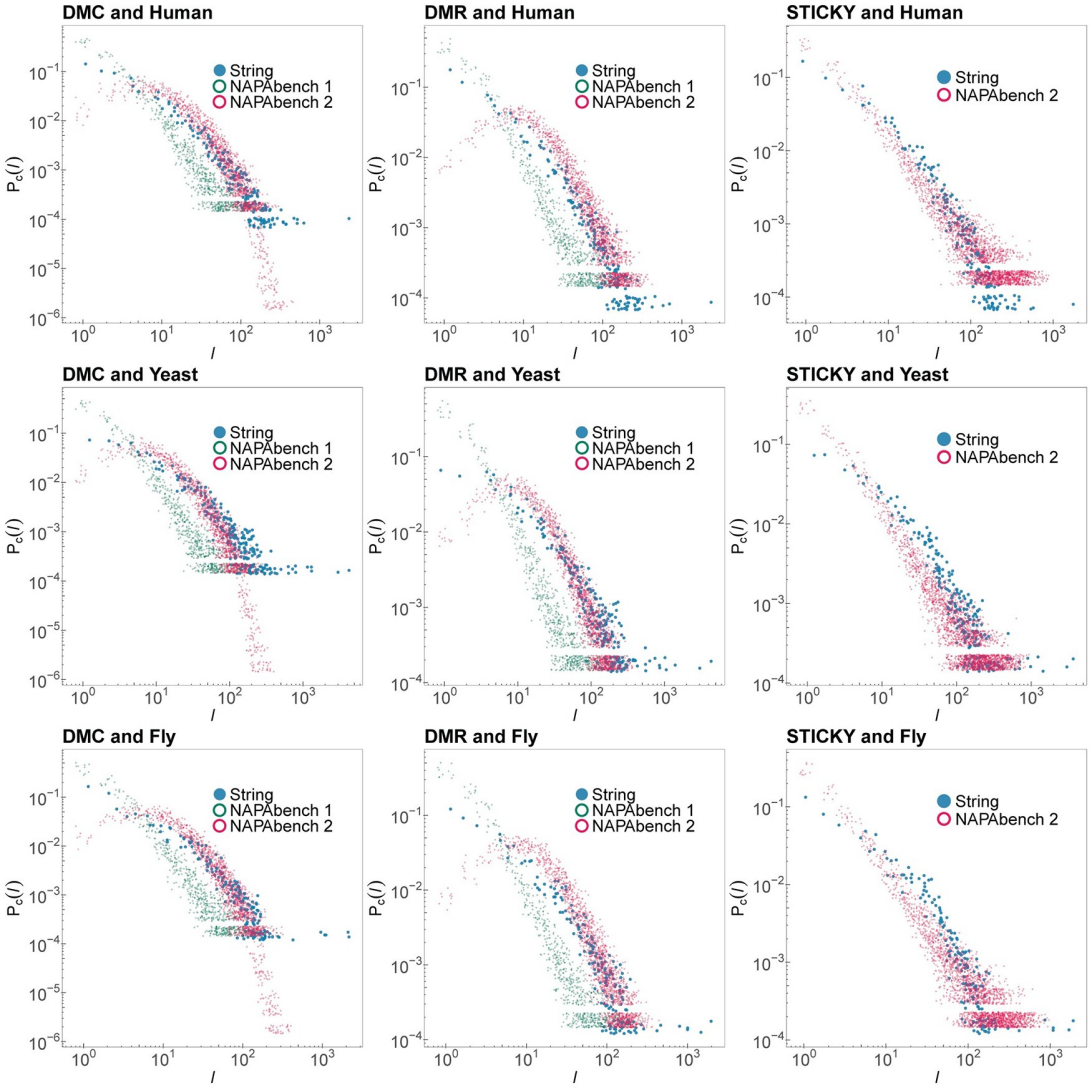
Node degree distribution comparison.

**Fig 9:**
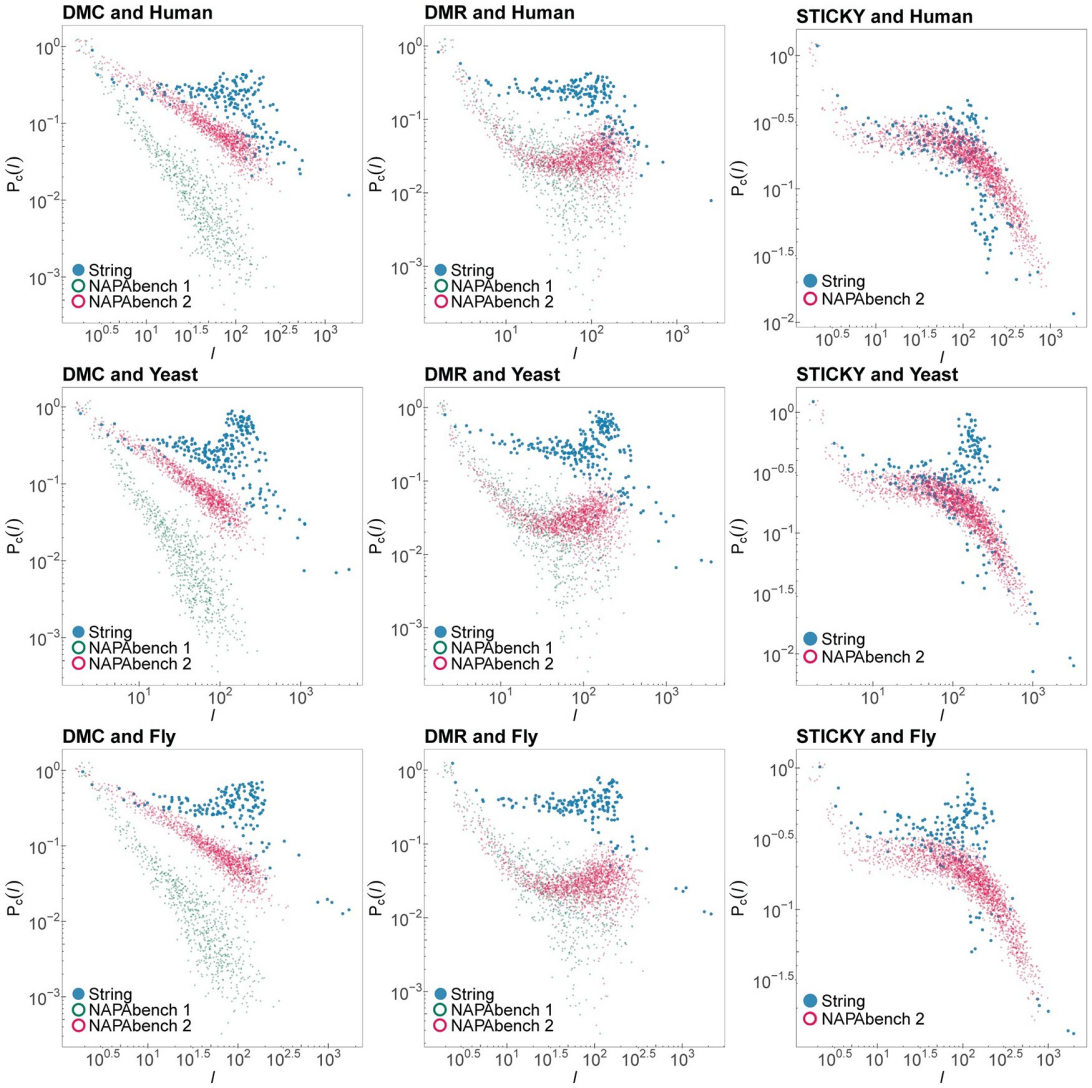
Clustering coefficient distribution comparison.

To further compare the capability of the network growth models to construct realistic PPI networks, we visualized the node degree distribution and the clustering coefficient distribution using scatter plots, which are shown in Figs 8 and 9. The scatter plots in Fig 8 compare the node degree distributions resulting from the network growth models in NAPAbench 1 and those resulting from the models in NAPAbench 2. For comparison, the node degree and clustering coefficient distributions are also shown for the three real PPI networks. In these plots, we used the first 10 out of the 100 realizations that were previously generated to obtain the results in Table 2, to avoid overcrowding the plots. As we can see, the red-colored circles corresponding to NAPAbench 2 networks overlap fairly well with the blue dots that correspond to real PPI networks. The scatter plots also clearly show the statistical deviation between the node degree distributions resulting from the network growth models in NAPAbench 1 (depicted in green circles) and the distributions observed in real PPI networks. Fig 8 clearly shows that the updated network growth models in NAPAbench 2, including the newly added STICKY model, are capable of generating PPI networks whose node degree distributions are statistically similar to those of the real PPI networks. Similarly, Fig 9 compares the cluster coefficient distributions that result from the network growth models in NAPAbench 1 and 2 with the distributions obtained from the three real PPI networks. The scatter plots in Fig 9 show that the updated network growth models in NAPAbench 2 consistently improve the clustering coefficient distributions compared to the NAPAbench 1 network growth models, bringing them closer to the distributions observed in real PPI networks. Especially, the clustering coefficients of the networks generated by the extended STICKY model (shown in the third column of Fig 9) closely resembled those in real PPI networks, in terms of their distributions.

**Fig 10:**
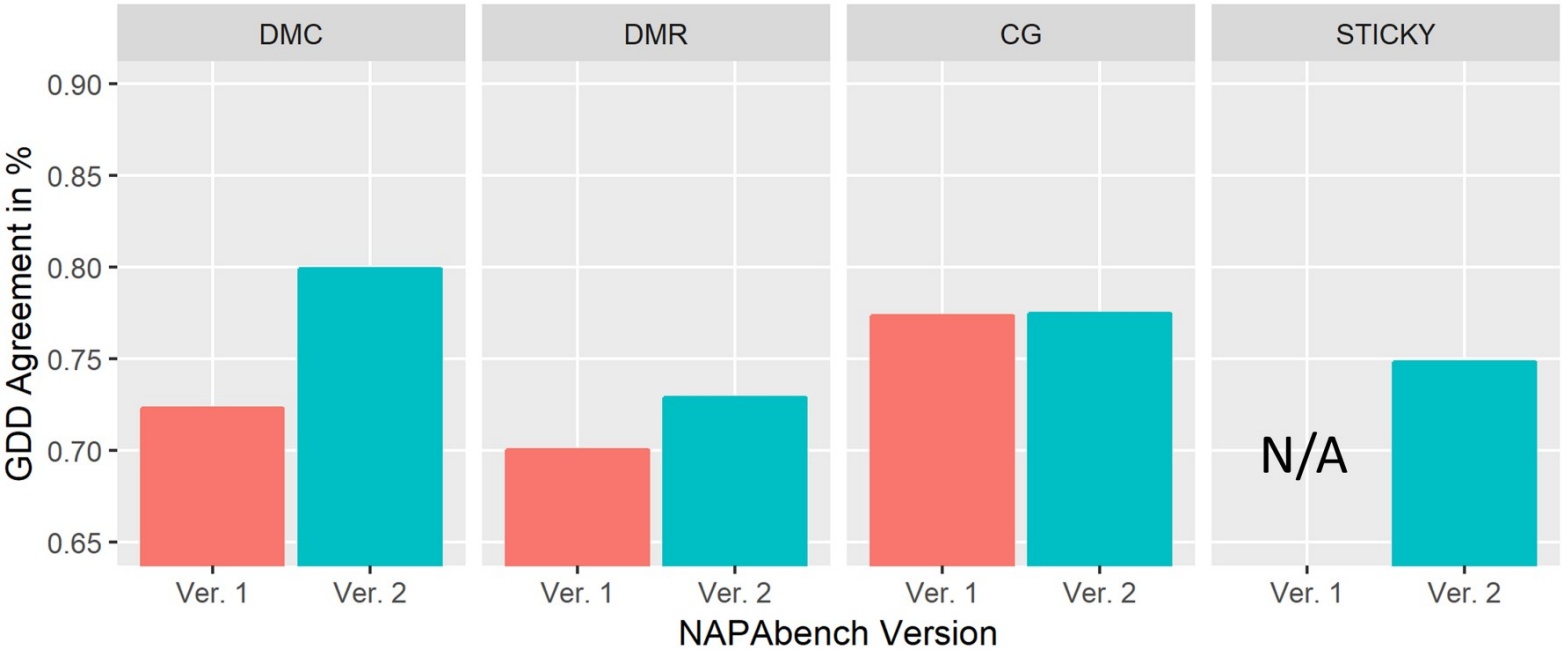
Graphlet degree distribution agreement (GDDA) with the yeast PPI network in STRING.

For additional statistical comparison between the PPI networks generated by the network growth models in NAPAbench 1 and NAPAbench 2, we computed the GDDA (graphlet degree distribution agreement) score between the yeast PPI network and a synthetic PPI network generated by a specific network growth model. Fig 10 shows that the networks synthesized by the network growth models in NAPAbench 2 achieved higher GDDA scores compared to those synthesized by NAPAbench 1 models.

The statistical comparison results in Table 2 and Figs 8–10 clearly show that the updated network models in NAPAbench 2 are capable of synthesizing more realistic PPI network families, whose topological characteristics closely resemble those of the latest real PPI networks, compared to the previous models in NAPAbench 1. Further statistical validation demonstrates that the NAPAbench 2 models produce more realistic networks compared to NAPAbench 1 models whose characteristics match those of real networks more closely. These results can be found in the supplementary material.

### Updated network alignment performance assessment benchmark: NAPAbench 2

**Fig 11:**
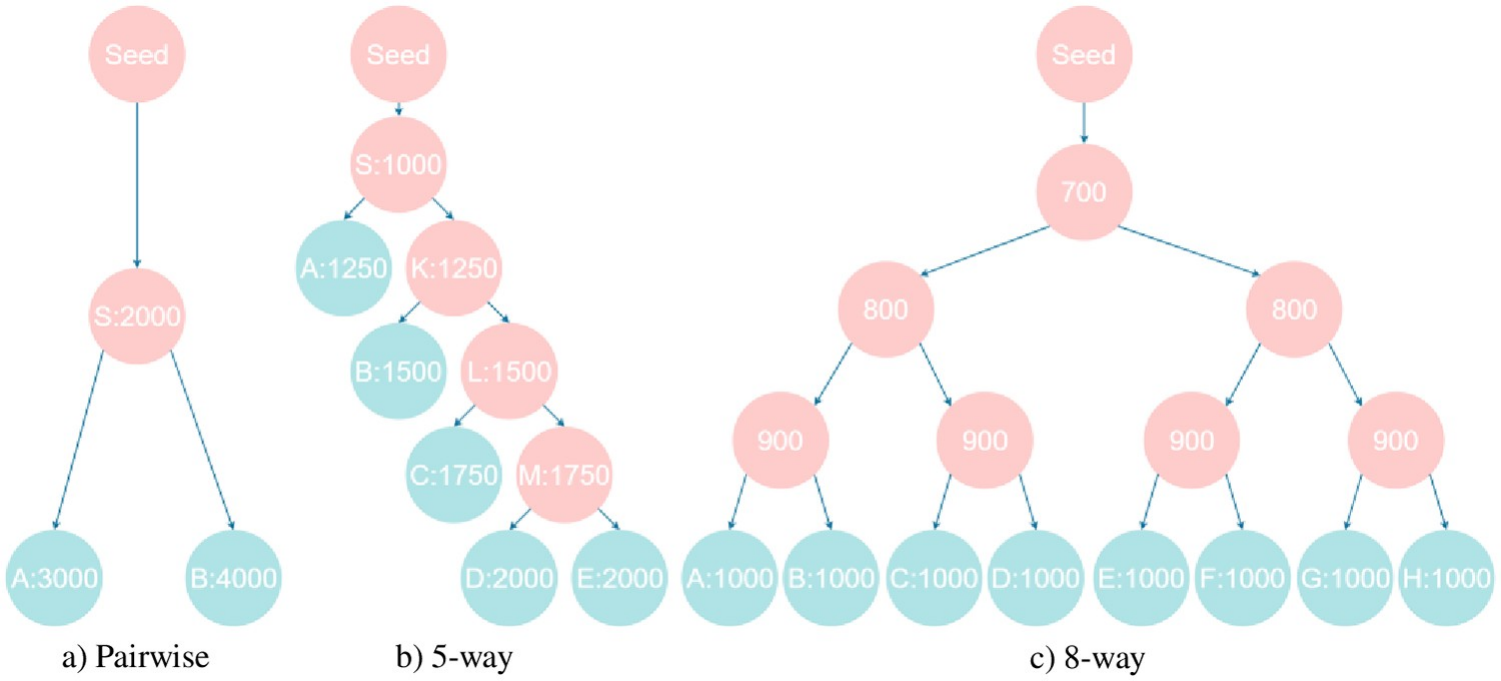
Phylogenetic trees adopted in NAPAbench 2.

We generated three suites of datasets, pairwise, 5-way, and 8-way according to the three pre-defined phylogenetic trees as shown in Fig 11. Note that each node in Fig 11 indicates individual synthetic PPI network and the number in the node represents the total number of proteins in the synthetic network. The mint-colored nodes stand for output synthetic PPI networks of NAPAbench 2, and pink colored nodes are ancestral or internal networks that are not included in the generated benchmark dataset. In NAPAbench 2, each suite includes ten network families per network growth model. In pairwise dataset (Fig 11a), each family consists of a network pair $G_{A}$ with *N*_*A*_ = 3, 000 and $G_{B}$ with *N*_*B*_ = 4, 000. Both networks are evolved from an ancestral network $G_{S}$ with *N*_*S*_ = 2, 000. In 5-way dataset (Fig 11b), each family contains five PPI networks with 1, 250, 1, 500, 1, 750, 2, 000, and 2, 000 nodes, respectively. Each family from 8-way dataset (Fig 11c) has eight PPI networks containing 1, 000 nodes, respectively.

### Potential limitations of the current network growth models in NAPAbench 2

Although NAPAbench 2 comes with updated network growth models that can synthesize realistic networks, whose properties closely resemble those of the latest real PPI networks, there is still room for further improvement. One potential limitation of the current approach lies in how functionalities are assigned to the nodes (corresponding to proteins) in the synthesized networks. Several studies have pointed out that interaction patterns of proteins often define the functionality of the proteins, resulting in the hierarchical functional structure of proteins [41, 42]. Furthermore, there exist special proteins called multi-functional proteins, such as Transglutaminase 2 (TG2) [43] or Ribosomal protein S3 (RPS3) [44], that can change their functions and interaction patterns under different conditions. However, the current network growth models in NAPAbench 2 do not consider the aforementioned properties. Another potential limitation of NAPAbench 2 is that it does not currently support the combined utilization of multiple network growth models for synthesizing the networks. As the individual network growth models utilized in NAPAbench 2 have low degrees of freedom, it is practically challenging to optimize their parameters to make the synthetic networks resemble real PPI networks based on multiple criteria. Combining multiple network growth models for network synthesis may give rise to more sophisticated models that may potentially generate more realistic networks.

## Concluding remarks

In this paper, we present NAPAbench 2, a comprehensive update to the original NAPAbench [2] that was originally released in 2012. NAPAbench 2 provides a network synthesis algorithm with an intuitive and user-friendly GUI that can be used to generate biologically realistic PPI network families, whose properties closely match those of the latest PPI networks in STRING v10.0 [16]. Furthermore, this new release includes a comprehensive network alignment benchmark that consists of 120 network families comprised of 600 networks. The new benchmark enables objective performance assessment of network alignment algorithms based on synthetic network families whose characteristics are similar to the latest PPI networks and for which the ground truth alignment is known. The accompanying network synthesis tool could be easily used to generate further benchmarks—for example, families that consist of a very large number of genome-scale networks—to assess the scalability and efficacy of network alignment algorithms.

## Supporting information

S1 Appendix Supplementary material for “NAPAbench 2: A network synthesis algorithm for generating realistic protein-protein interaction (PPI) network families”. (PDF)
