# Supplementary material for: NAPAbench 2: A network synthesis algorithm for generating realistic protein-protein interaction (PPI) network families
Source: PLoS One. 2020 Jan 27;15(1):e0227598. doi: 10.1371/journal.pone.0227598 (PMC6984706; doi:10.1371/journal.pone.0227598)
Supplement: S1 Appendix — (PDF) [file pone.0227598.s001.pdf]

Supplementary Material for

"NAPAbench 2: a network synthesis algorithm for generating realistic protein-protein interaction (PPI) network families"

Hyun-Myung Woo[1¶], Hyundoo Jeong[2¶], Byung-Jun Yoon[1,3,4*]

1 Department of Electrical and Computer Engineering, Texas A&M University, College Station, TX, USA

2 Department of Mechatronics Engineering, Incheon National University, Incheon, Republic of Korea

3 TEES-AgriLife Center for Bioinformatics and Genomic Systems Engineering, Texas A&M University, College Station, TX, USA

4 Computational Science Initiative, Brookhaven National Laboratory, Upton, NY, USA

*** Corresponding author**

E-mail: bjyoon@ece.tamu.edu

¶ These authors contributed equally to this work.

S1 Instruction

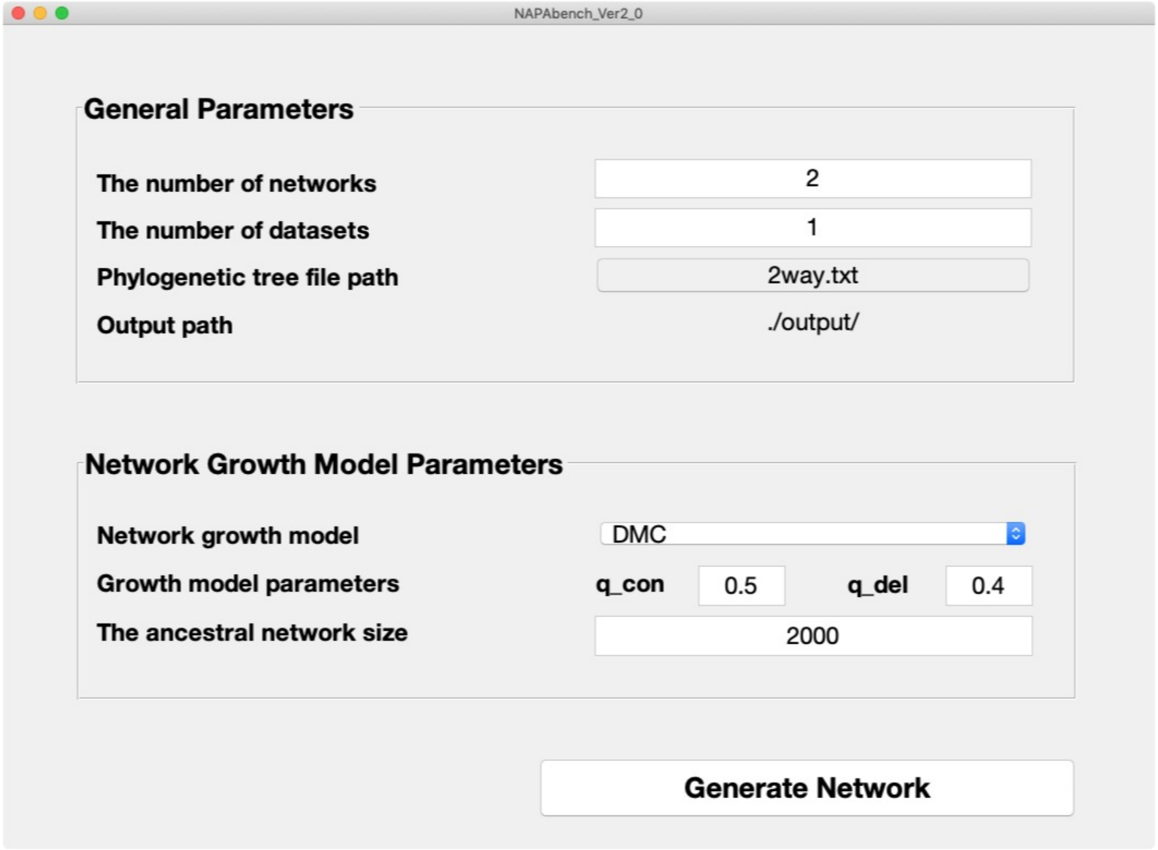

Figure S1. Graphical user interface (GUI) of NAPAbench 2 network synthesis tool.

The network synthesis algorithm that is included in NAPAbench 2 comes with a user-friendly graphical user interface (GUI) (see Fig. S1). A user may tune parameters to generate a synthetic PPI network benchmark dataset if needed. We also provide default setups for pairwise, 5-way, and 8-way dataset with phylogenetic tree files, which have been used to construct NAPAbench 2. The default setup for pairwise, 5-way, and 8-way will be automatically applied when a user enters 2, 5, or 8 for the number of networks, respectively.

Input

1. General Parameters

   (a) The number of networks: the number of synthesized networks. The number of networks is

equal to the number of leaf nodes in a phylogenetic tree.

(b) The number of datasets: the number of samples. It is equal to the number of families in the output benchmark dataset.

(c) Phylogenetic tree file path: a user may modify the phylogenetic tree using text input and load it to NAPAbench 2 program. For more information, please refer to the following sections.

(d) Output path: It describes the path for output generated by NAPAbench 2 program.

2. Network Growth Model Parameters

   (a) Network growth model: a user may freely select a network growth model to develop networks in the phylogenetic tree.

   (b) Growth model parameters: each network growth model has its own parameters as follows. For more information, please refer to the following sections.

   (c) The ancestral network size: the number of nodes in the ancestral node $\mathcal{G}_S$.

Output

1. log_file.txt: It describes options used to generate a dataset, and statistics of output.

2. X.net: PPI network for each node with respect to the input phylogenetic tree. We utilized a tab-delimited format so two proteins with interactions are placed in the same line.

3. X.fo: List of functional orthology annotation of proteins.

4. X–Y.sim: Pairwise node similarity.

Tuning parameters

1. **Phylogenetic tree file**

   A user may modify a phylogenetic tree file which is described by Markov language. An invalid description may lead to incorrect output. There are several rules regarding the phylogenetic tree file as follows.

   (a) The phylogenetic tree should be a full binary tree meaning each node should have either two of zero ancestral nodes.

(b) All nodes should be labeled.

(c) The number of nodes in descendant nodes should be equal to or greater than the number of nodes in a parent node.

(d) Lines starting with “//” will be commented.

(e) Description format: {parent network ID}->“{descendent network ID}:{number of nodes in the descendent network}

Example: Representation of a phylogenetic tree is shown in Fig. S2 for a network family comprising five networks.

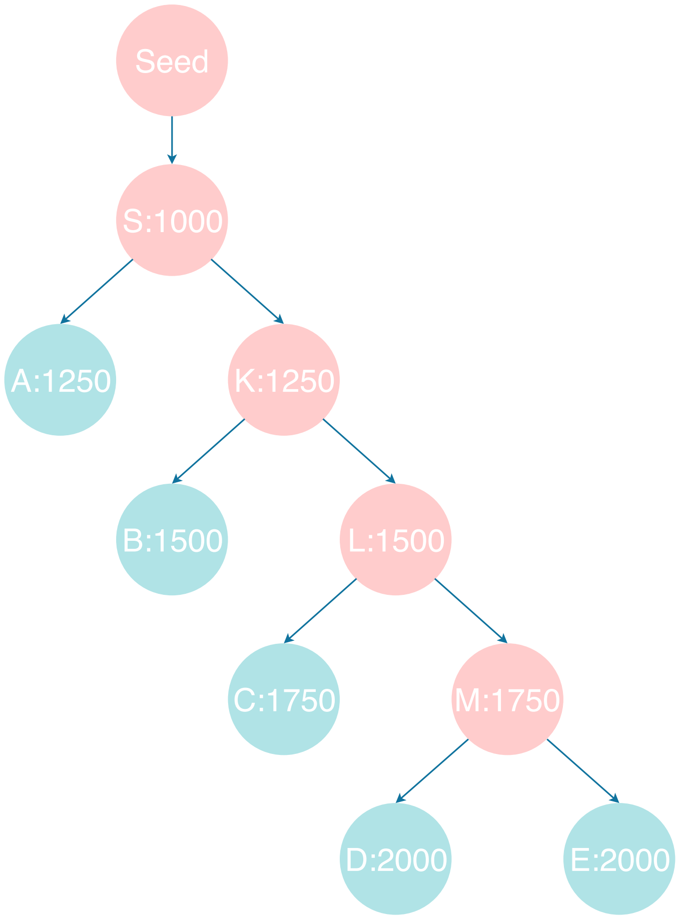

Figure S2. Illustration of 5-way phylogenetic tree.

```
S->A:1250
S->K:1250
K->B:1500
K->L:1500
L->C:1750
L->M:1750
M->D:2000
M->E:2000
```

2. **Modifying network growth models**

   A user may modify properties of the synthetic PPI networks by tuning parameters of network growth models. The number of PPIs can be increased by tuning the parameters as follows.

   - DMC model: Increasing q_{con}, or decreasing q_{mod} (Higher impact).
   - DMR model: Increasing q_{new}, or decreasing q_{del} (Higher impact).
   - CG model: Increasing δ.
   - STICKY model: Decreasing s_{del}.

   Note that tuning q_{mod}, q_{del}, δ, and s_{del} will lead to huge changes in statistics of PPI network output. For algorithms controlled by more than one parameter, it is recommended to tune one parameter at a time.

S2 Statistical significance test results comparing the network growth models in NAPAbench version 1 and version 2

We performed quantitative validations and comparisons for the statistical features of the synthetic networks generated by each network growth model under different parameter settings in NAPAbench 2 and NAPAbench 1. Note that we did not perform statistical validations for CG and STICKY models because STICKY model was not included in the original NAPAbench and as the CG model has not been updated in the updated NAPAbench (version 2). For DMC and DMR models, we generated 100 synthetic networks based on the tailored model parameters in NAPAbench version 1 and version 2, respectively.

Table S1. Kolmogorov-Smirnov normality test for the degree exponent, average node degree, and GDDA score.

	DMC		DMR	
	NAPAbench 1	NAPAbench 2	NAPAbench 1	NAPAbench 2
Degree exponent	5.196×10^{-45}	2.655×10^{-27}	2.130×10^{-43}	1.410×10^{-25}
Average node degree	0	1.403×10^{-23}	0	1.057×10^{-12}
GDDA score	2.942×10^{-12}	1.255×10^{-13}	3.488×10^{-12}	1.178×10^{-12}

Table S2. Mann-Whitney significance test results for evaluating the statistical significance of the differences between networks generated by network growth models in NAPAbench 1 and those in NAPAbench 2.

	DMC	DMR
Degree exponent	$1.3604 \times 10^{-34} \ll 0.05$	$1.2811 \times 10^{-34} \ll 0.05$
Average node degree	$1.281 \times 10^{-34} \ll 0.05$	$1.281 \times 10^{-34} \ll 0.05$
GDDAscore	$3.398 \times 10^{-8} \ll 0.05$	$4.146 \times 10^{-5} \ll 0.05$

Given the 100 synthetic networks for each network growth model, we estimated three topological features of the synthetic networks: i) degree exponent, ii) average node degree, and iii) GDDA scores. Note that GDDA scores were obtained by comparing the synthetic networks and the yeast PPI network, and we utilized randomly sampled 20 synthetic networks because computing GDDA scores requires a longer computation time. Then, the degree exponent and average node degree are transformed into the relative measurement by subtracting each measurement from that of the yeast PPI network. This approach allows us to validate the statistical significance of the structural differences between synthetic networks in NAPAbench 2 and NAPAbench 1 with respect to the reference yeast PPI network. Since the GDDA score can be obtained by comparing two networks and it is a relative metric itself, we directly utilized the GDDA scores to validate the statistical difference between the synthetic networks without further processing.

We first examined the distribution of the topological features to find out whether they follow the normal distribution. To this aim, we adopted the Kolmogorov-Smirnov normality test. Table S1 provides strong evidence that the topological features do not follow the normal distribution, hence Students t-test may not be appropriate for showing the statistical differences between synthetic networks generated by models in NAPAbench 1 and 2. Instead, we performed the Mann-Whitney rank test to statistically validate the topological differences between the synthetic networks generated by NAPAbench 1 models and those generated by NAPAbench 2 models. As we can see in Table S2, NAPAbench 1 models and NAPAbench 2 models generate networks with different topological features with high statistical significance.

The statistical comparisons included in the main paper (see Fig 8–10 and Table 2) and the above statistical significance test results clearly show that NAPAbench 2 network growth models generate better (*i.e.*, more realistic) synthetic networks compared to NAPAbench 1 models, where the differences between the networks resulting from the different models are shown to be statistically significant when tested based on 100 random networks.
